# Pathogenesis and therapeutic strategies in the interaction between gut microbiota and enterohemorrhagic *Escherichia coli* infection

**DOI:** 10.1093/femsre/fuag045

**Published:** 2026-08-31

**Authors:** Xueping Li, Di Huang, Ziwei Chen, Luna Cui, Sheng Wang, Tao Wang, Bin Liu, Yutao Liu

**Affiliations:** School of Life Sciences, Faculty of Medicine, Tianjin University, Tianjin 300072, China; State Key Laboratory of Synthetic Biology, Tianjin University, Tianjin 300072, China; National Key Laboratory of Intelligent Tracking and Forecasting for Infectious Diseases, TEDA Institute of Biological Sciences and Biotechnology, Nankai University, Tianjin 300457, China; The Key Laboratory of Molecular Microbiology and Technology, Ministry of Education, Nankai University, Tianjin 300071, China; Nankai International Advanced Research Institute, Nankai University, Shenzhen, Shenzhen 518045, China; School of Environmental Science and Engineering, Tianjin University, Tianjin 300072, China; School of Life Sciences, Faculty of Medicine, Tianjin University, Tianjin 300072, China; State Key Laboratory of Synthetic Biology, Tianjin University, Tianjin 300072, China; School of Life Sciences, Faculty of Medicine, Tianjin University, Tianjin 300072, China; State Key Laboratory of Synthetic Biology, Tianjin University, Tianjin 300072, China; School of Life Sciences, Faculty of Medicine, Tianjin University, Tianjin 300072, China; State Key Laboratory of Synthetic Biology, Tianjin University, Tianjin 300072, China; National Key Laboratory of Intelligent Tracking and Forecasting for Infectious Diseases, TEDA Institute of Biological Sciences and Biotechnology, Nankai University, Tianjin 300457, China; The Key Laboratory of Molecular Microbiology and Technology, Ministry of Education, Nankai University, Tianjin 300071, China; Nankai International Advanced Research Institute, Nankai University, Shenzhen, Shenzhen 518045, China; National Key Laboratory of Intelligent Tracking and Forecasting for Infectious Diseases, National Center for Infectious Diseases, Beijing Institute of Infectious Diseases, Beijing Ditan Hospital, Capital Medical University, Beijing 100015, China

**Keywords:** enterohemorrhagic *Escherichia coli*, gut microbiota, microbiota–derived metabolites, virulence regulation, host-microbe interactions, probiotic and prebiotic interventions

## Abstract

Enterohemorrhagic *Escherichia coli* (EHEC) is a major foodborne pathogen that causes hemorrhagic colitis and hemolytic uremic syndrome. Increasing evidence indicates that the gut microbiota plays a central role in modulating EHEC pathogenesis through complex metabolic and signaling networks. Beneficial commensals, including *Bifidobacterium, Lactobacillus*, and segmented filamentous bacteria, contribute to colonization resistance by competing for nutrients and adhesion sites, producing antimicrobial metabolites, and enhancing epithelial barrier integrity. In contrast, certain species such as *Bacteroides thetaiotaomicron* and *Enterococcus faecalis* may promote EHEC virulence by altering intestinal nutrient availability or triggering the expression of virulence genes. Microbiota-derived metabolites, including short-chain fatty acids, succinate, indole, riboflavin, nicotinamide, ethanolamine, and L-malate, act as important regulatory signals that connect microbial metabolism with LEE-mediated virulence pathways. Understanding these host-microbe-pathogen interactions provides a mechanistic basis for developing microbiota-targeted interventions such as probiotics, prebiotics, and fecal microbiota transplantation that enhance colonization resistance and attenuate virulence. Integration of AI-based analytics with multi-omics approaches is expected to facilitate the design of personalized, mechanism-driven therapeutic strategies for the control of EHEC infection.

## Teaser

Microbiota-EHEC interaction in pathogenesis and therapy.

## Introduction

Enterohemorrhagic *Escherichia coli* (EHEC), a subtype of Shiga toxin-producing *E. coli* (STEC), represents a significant foodborne pathogen responsible for severe intestinal infections and constitutes a major global public health concern (Nataro,Kaper 1998, Chauret 2011). EHEC strains are classified according to their LPS(O) and flagellar (H) antigens. More than seven EHEC serogroups have been isolated from patients, including O26, O45, O103, O111, O121, O145, and the most common serotype, O157:H7 (Norman et al. 2012). Infections caused by EHEC O157:H7 can manifest in a spectrum of symptoms, ranging from mild watery diarrhea to severe hemorrhagic colitis (HC). In some cases, infection may progress to life-threatening complications such as hemolytic uremic syndrome (HUS), which typically presents with microangiopathic hemolytic anemia, thrombocytopenia, and acute kidney injury. However, the severity and combination of these clinical features may vary, and atypical presentations have also been reported. HUS remains a leading cause of acute renal failure in children (Herzog et al. 2023).

EHEC persists in various environments, including the intestines of healthy cattle and wildlife such as red foxes (Bertelloni et al. 2022). It can be transmitted through multiple routes, including contaminated foods (e.g. undercooked ground meat, leafy greens) and environmental sources (e.g. ponds, streams, well water) (Cherry 2022), where the bacterium can survive for months. Since emerging as a significant public health threat in the early 1980s, EHEC has consistently necessitated a public health response due to its wide range of clinical symptoms and the low infectious dose (around 10–100 cells) required to cause disease (Nguyen and Sperandio 2012). Further data indicate that EHEC infections account for an annual disease burden of over 265 000 illnesses (Nawrocki et al. 2020). North America and Europe have repeatedly documented large EHEC outbreaks with high morbidity, reinforcing EHEC as a leading community-acquired cause of HUS. These outbreaks frequently require hospitalization and can be fatal; during outbreaks, case-hospitalization proportions may approach or exceed 40%, with deaths reported (Barnett Foster 2013, Mengistu,Mengesha 2023).

During infection, EHEC is capable of sensing intestinal environment changes and regulating the expression of virulence genes, which are critical for successful bacterial colonization and pathogenesis (Sun et al. 2024a). Previous studies have shown that EHEC responds to host-derived cues such as magnesium concentration, hormone levels, pH, and mechanical forces to fine-tune virulence gene regulation (Liu et al. 2019, Ellermann et al. 2020, Liu et al. 2020, Sirisaengtaksin et al. 2020). Recent findings have further highlighted the role of the gut microbiota and its metabolites in modulating EHEC pathogenicity (Neumann et al. 2021). Beneficial microbes such as *Bifidobacterium* and *Lactobacillus* can exert protective effects by enhancing epithelial barrier integrity, competing for nutrients, and influencing host immune responses (Gopal et al. 2001, Putaala et al. 2008). In contrast, certain microbiota-derived compounds, including succinate, indole, and ethanolamine, not only serve as nutrient sources but also act as signaling molecules that either activate or repress EHEC virulence gene expression (Kendall et al. 2012, Kumar et al. 2019, Li et al. 2025). Thus, the ability of EHEC to integrate signals from the host and the gut microbiota establishes a complex and dynamic regulatory network that governs its pathogenic behavior. This environmental sensing capacity ensures that virulence gene expression is precisely coordinated with the intestinal milieu, ultimately shaping the course and outcome of infection.

The use of antibiotics in treating EHEC infections is a contentious topic due to concerns that they may increase the risk of HUS (Freedman et al. 2016). While antibiotics are typically the most common method to treat bacterial infections, their use in EHEC infections is contraindicated because they may induce the bacterial SOS response, leading to increased Shiga toxin production and release. This is because antibiotics can promote Shiga toxin production by enhancing the replication and expression of *stx* genes (Kumar et al. 2019). Further studies also support this contraindication, showing that children treated with antibiotics for HC associated with EHEC had an increased chance of developing HUS (Bielaszewska et al. 2012, Berger et al. 2019). Therefore, alternative strategies are needed to develop safe and effective antibacterial therapies to treat EHEC infection.

## The virulence factors in EHEC and *Citrobacter rodentium*

**Figure 1:**
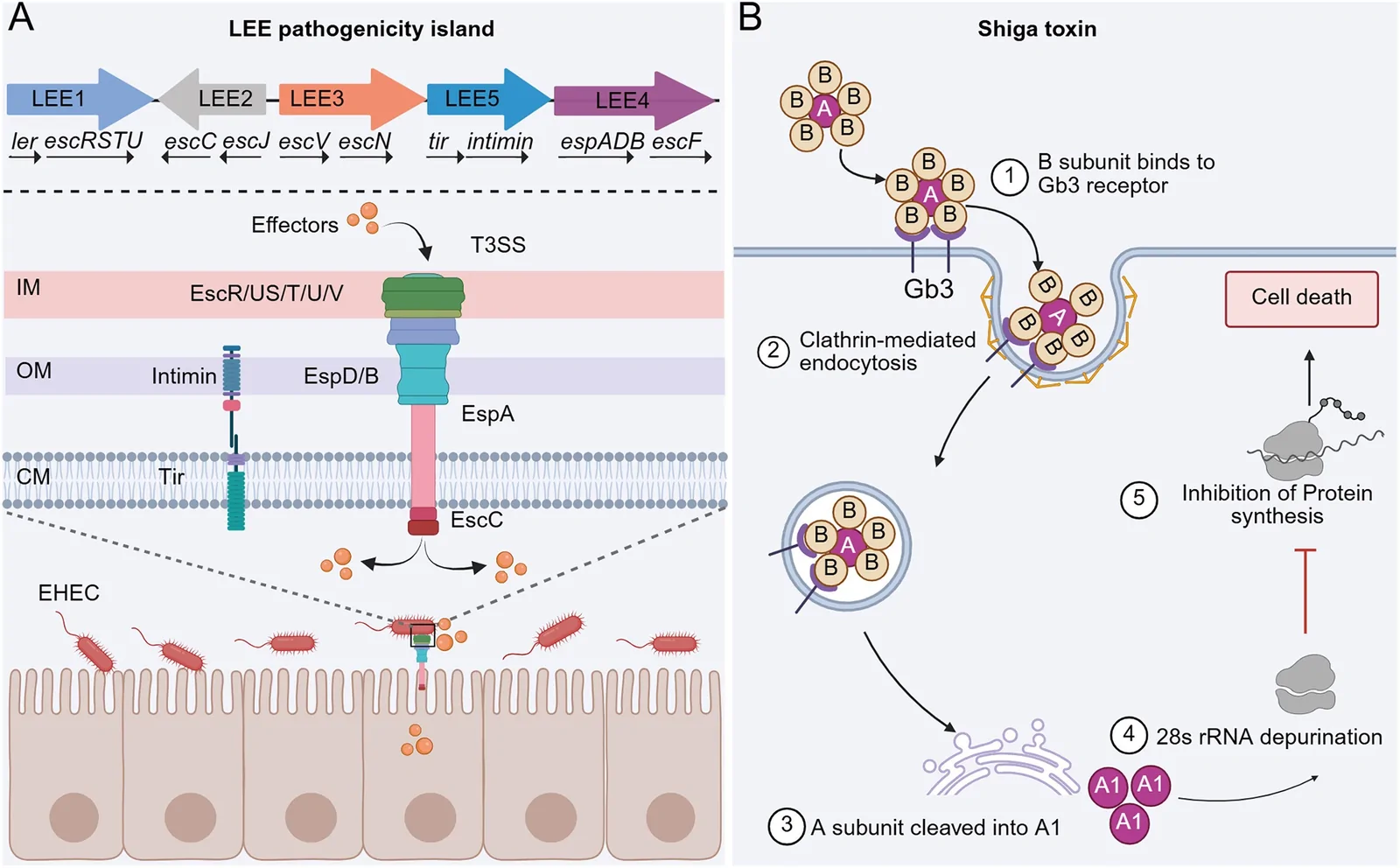
LEE pathogenicity island and Shiga toxin are key virulence factors of the EHEC. (A) The LEE pathogenicity island encodes a T3SS and most effectors required for the formation of A/E lesions. Schematic representation of the LEE pathogenicity island and its major operons (LEE1-LEE5). (B) Shiga toxin structure and pathogenic mechanism. Shiga toxin is an AB5-type toxin consisting of a catalytic A subunit non-covalently associated with a pentameric receptor-binding B subunit. IM, inner membrane; OM, outer membrane; CM, cell membrane; T3SS, type III secretion system; Tir, translocated intimin receptor; Gb3, globotriaosylceramide.

The pathogenesis of enterohemorrhagic *E. coli* (EHEC) relies on two major virulence determinants: the production of Shiga toxins (Stx1 and Stx2) and the ability to form attaching and effacing (A/E) lesions on intestinal epithelial cells (Ritchie et al. 2003). After ingestion, EHEC survives passage through the stomach and small intestine before colonizing the large intestine, where it adheres tightly to the epithelial surface. The formation of A/E lesions is driven by genes located on the locus of enterocyte effacement (LEE) pathogenicity island, which encodes the type III secretion system (T3SS) and its associated effectors (Franzin,Sircili 2015). The LEE comprises five operons (LEE1–LEE5) (McDaniel et al. 1995). LEE1 encodes Ler, the central transcriptional activator that controls the expression of the other LEE operons (McDaniel et al. 1995). LEE2 and LEE3 specify structural components of the T3SS apparatus, whereas LEE4 encodes the translocators and additional effectors required for protein injection into host cells. LEE5 produces two critical adhesion molecules—intimin, an outer membrane adhesin, and Tir, its translocated receptor (Garmendia et al. 2005). Once delivered into the host cell membrane, Tir interacts with intimin to establish intimate bacterial attachment (Frankel et al. 1998). Additional effector proteins, including EspF and EspG, remodel the actin cytoskeleton, disrupting microvilli and generating the characteristic pedestal structures of A/E lesions. These orchestrated events enable EHEC to colonize the colonic epithelium and contribute to its pathogenic outcomes (Fig. 1), (Hardwidge et al. 2005, Hua et al. 2018).

Shiga toxin (Stx) is another key virulence factor produced by EHEC. This toxin is encoded by phage genes integrated into the EHEC genome and belongs to the AB5 toxin family, consisting of a single enzymatic A subunit non-covalently bound to a pentameric B subunit (Boisen et al. 2015). The B subunit mediates toxin internalization by binding to Globotriaosylceramide(Gb3) receptors of host endothelial cells, particularly in the kidneys. Once inside the cell, Shiga toxin is transported to the endoplasmic reticulum (ER). Then, the A subunit is cleaved into an enzymatically active A1 fragment, which acts as an RNA N-glycosidase, removing an adenine residue from 28S rRNA, thereby interrupting protein synthesis and causing cell death (Melton-Celsa 2014). This mechanism leads to renal endothelial damage, thrombocytopenia, and microangiopathic hemolytic anemia, ultimately manifesting as HC and HUS (Fig. 1), (Melton-Celsa 2014).

*Citrobacter rodentium* (*C. rodentium*) is a Gram-negative, mouse-restricted enteric pathogen that causes transmissible colonic hyperplasia and A/E lesions in the murine intestinal epithelium (Collins et al. 2014). Like EHEC, *C. rodentium* harbors the conserved LEE pathogenicity island encoding the type III secretion system (T3SS), intimin, Tir, and numerous effectors required for lesion formation and host cytoskeletal remodeling (Deng et al. 2001). As a natural murine pathogen, it reliably colonizes immunocompetent mice and enables mechanistic investigation of bacterial colonization, LEE-dependent virulence, epithelial barrier disruption, mucosal immunity, and the contribution of the gut microbiota under physiologically relevant *in vivo* conditions. Therefore, this model has been extensively used to define signaling pathways and microbial metabolites that regulate the virulence of attaching and effacing pathogens (Deng et al. 2001, Petty et al. 2010).

Nevertheless, important limitations should be considered when extrapolating findings from this model to human EHEC infection. Unlike EHEC, *C. rodentium* does not encode Shiga toxin and does not reproduce the toxin-mediated systemic complications of EHEC infection, including hemorrhagic colitis, renal endothelial injury, and hemolytic uremic syndrome (Richardson et al. 1988, Deng et al. 2003, Psotka et al. 2009, Petty et al. 2010, Obrig and Karpman 2012). In immunocompetent mice, *C. rodentium* infection generally causes transient colonic inflammation and hyperplasia rather than the severe intestinal and renal disease observed in humans with EHEC infection. Thus, the model is particularly valuable for studying conserved LEE-mediated colonization and microbiota-dependent mechanisms. However, findings should be interpreted cautiously when considering Shiga toxin-associated systemic disease and human-specific host-pathogen interactions.

To overcome this limitation, several animal models have been employed to study EHEC pathogenesis. First, infant rabbits have been used to model EHEC infection, as they reproduce bacterial adherence and some hallmarks of disease in a physiologically relevant host (Warr et al. 2021). In parallel, many of the signaling pathways and virulence factors of EHEC have been elucidated using the *C. rodentium* mouse infection model, which serves as a convenient surrogate for studying the mechanisms of A/E pathogens. Moreover, to better recapitulate EHEC-associated systemic disease, investigators engineered a Shiga toxin–producing *C. rodentium* by lysogenizing it with an EHEC-derived Stx2 phage (ϕStx2dact). Upon SOS-driven prophage induction, *C. rodentium* (ϕStx2dact) produces Stx2 at levels comparable to human EHEC isolates and, following oral infection of microbiota-replete C57BL/6 mice, causes toxin-dependent weight loss, diarrhea, colonic inflammation, renal pathology, and proteinuria (Flowers et al. 2016, Thorpe et al. 2021).

## Complex relationship between microbiota and EHEC infection

The host intestine contains a complex and dynamic microbiota that plays crucial roles in nutrient metabolism, energy regulation, and immune development (Theodorou et al. 2014). Recent research has increasingly focused on the interactions between the microbiota and pathogens, demonstrating that the interactions between the microbiota and EHEC are far more complex.

**Figure 2:**
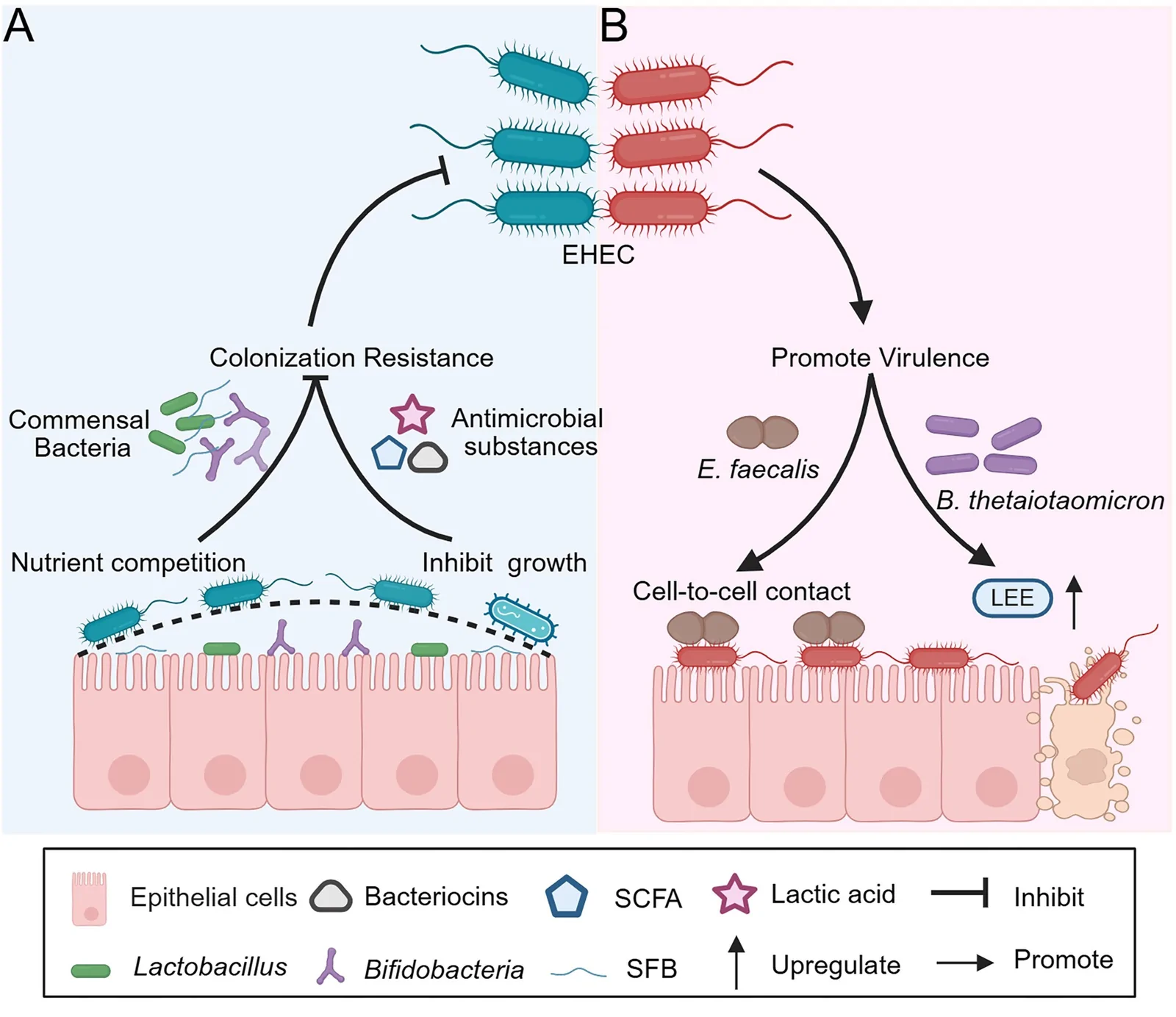
Complex interactions between the gut microbiota and EHEC. The intestinal microbiota exerts both protective and promoting effects on EHEC colonization. (A) Commensal bacteria, such as *Lactobacillus* and *Bifidobacteria*, contribute to colonization resistance by competing for nutrients and inhibiting EHEC growth. (B) Certain commensals such as *Bacteroides thetaiotaomicron, Enterococcus* species can enhance EHEC virulence through multiple pathways. These opposing influences illustrate the complex and dynamic nature of microbiota–EHEC interactions that determine infection outcomes.

A key defensive mechanism of the microbiota against intestinal pathogens is colonization resistance, which is the ability of the commensal gut microbiota to prevent the colonization and overgrowth of intestinal pathogens (Jacobson et al. 2018). Under normal conditions, the microbiota occupies the ecological niches in the intestine and competes with pathogens for nutrients and attachment sites. For instance, beneficial bacteria like *Bifidobacteria* efficiently utilize diverse carbohydrates released from dietary fibers or mucin, leaving limited resources available for invading pathogens. This nutrient competition is enhanced by the production of antimicrobial substances, including bacteriocins, lactic acid, and SCFAs, which help lower the pH and inhibit EHEC growth (Pokusaeva et al. 2011). In addition, these metabolites such as SCFAs, amino acids, bile acids, and vitamins can enhance the integrity of the intestinal barrier by promoting tight junction formation and stimulating the mucus production by goblet cells (Fig. 2), (Chandrasekaran et al. 2024).

Contrasting the protective role of colonization resistance, certain members of the microbiota can promote the virulence of EHEC under specific situations. One example is *Bacteroides thetaiotaomicron* (*B. thetaiotaomicron*), a predominant gut commensal that can degrade mucus and alter the available fucose levels in the colon. The change of fucose levels can serve as a signal that triggers EHEC virulence genes expression, which facilitates the attachment to intestinal epithelial cells and the formation of A/E lesions(Pacheco et al. 2012). Similarly, other commensals, such as *Enterococcus* species, have been reported to modulate EHEC pathogenicity through mechanisms that require direct cell-to-cell contact, as discussed in detail in Section 4.5 (Cameron et al. 2019). These observations demonstrate that when specific commensals are overgrowth, the gut environment may be preferred for EHEC survival and virulence gene expression (Fig. 2).

In conclusion, the gut microbiota and EHEC are engaged in an evolutionary arms race, wherein each adapts to the changing environmental cues and pressures imposed by the other. Recent *in vitro* and *in vivo* studies have shown that the interactions are far more complex than protection and promotion. In this review, we will discuss in detail the various interaction patterns between different microbiota and EHEC.

## Microbiota and EHEC infection

### Bifidobacterium

*Bifidobacterium* is a genus of Gram-positive, anaerobic bacteria naturally resident in the human intestine and contributes to host metabolism via saccharolytic fermentation of glycans that are abundant in the proximal colon (Riviere et al. 2016). *Bifidobacteria* play critical roles in maintaining the balance of the gut environment, which contributes to host health through the competitive exclusion of pathogenic bacteria, modulation of local pH, and enhancement of mucosal integrity (Tojo et al. 2014, Srutkova et al. 2015). Available evidence indicates that the effects are strain specific and depend on bacterial metabolic traits, the host background, and the experimental infection model.

**Figure 3:**
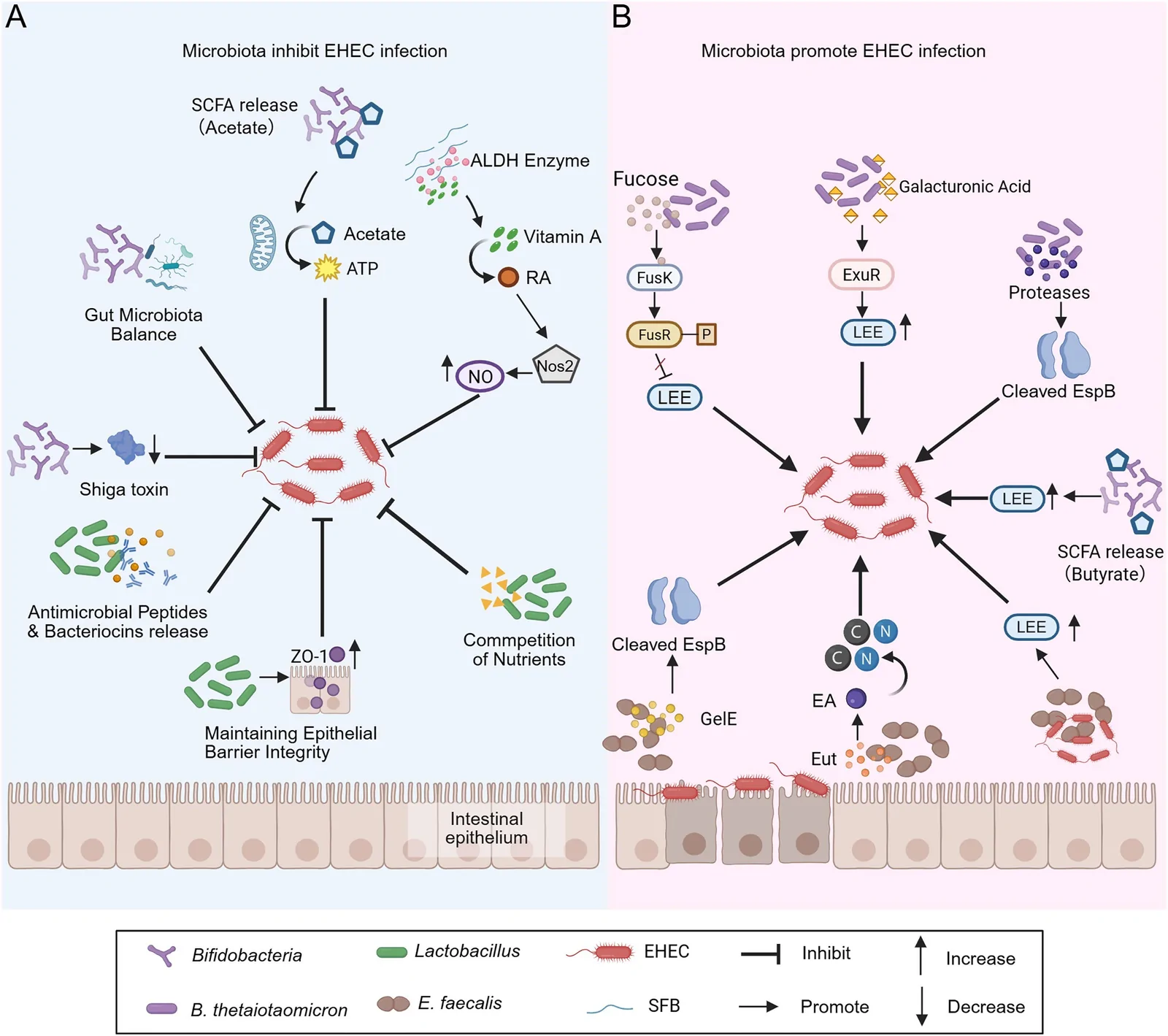
Mechanisms by which gut microbiota inhibits or promotes EHEC infection.(A) The gut microbiota (*Bifidobacteria*, SFB, *Lactobacillus*) protects its host from pathogens via mechanisms including the production of short-chain fatty acids (SCFAs and bacteriocins, the conversion of primary into secondary bile salts, the competition for nutrients from the host diet, maintenance of epithelial barrier function, and the inhibition of the regulation of the LEE genes. (B) Gut commensal bacteria (*Bacteroides thetaiotaomicron, Enterococcus faecalis*) enhance EHEC virulence and colonization through the production of specific metabolites (fucose, galacturonic acid, EA) that act as signals regulating virulence gene expression or as nutrients facilitating pathogen expansion. SFB, segmented filamentous bacteria; EA, ethanolamine; SCFAs, short-chain fatty acids.

Direct evidence from a streptomycin-treated mouse model of STEC O157:H7 infection illustrates the specificity of these strains. Oral administration of *Bifidobacterium breve* strain Yakult or *Bifidobacterium pseudocatenulatum* DSM 20439 prevented mortality and significantly reduced intestinal concentrations of Stx1 and Stx2(Asahara et al. 2004). By contrast, *Bifidobacterium bifidum* ATCC 15696 and *Bifidobacterium catenulatum* ATCC 27539^T^ did not confer protection, although all four strains achieved similar intestinal population levels(Asahara et al. 2004). Therefore, protection could not be explained simply by successful intestinal colonization. The two protective strains were associated with reduced caecal pH, increased total organic-acid and acetate concentrations, and reduced succinate concentrations. Under in vitro conditions mimicking the intestinal environment of mice colonized with *B. breve* strain Yakult, the combined lower pH and higher acetate concentration markedly reduced Stx2 production, including mitomycin C-induced Stx2 production, without appreciably altering STEC growth (Asahara et al. 2004). These results suggest that strain-dependent modulation of the intestinal metabolic environment can reduce toxin production (Fig. 3).

Mechanistic studies in gnotobiotic mice have further identified metabolic and genetic determinants of protection. *Bifidobacterium longum subsp. longum* JCM 1217^T^, *B. longum subsp. longum* NCC2705, and *B. longum subsp. infantis* 157F-4–1 protected mice against lethal EHEC O157:H7 infection, whereas *Bifidobacterium adolescentis* JCM 1275^T^ and *Bifidobacterium thermacidophilum* JCM 1207^T^ did not (Fukuda et al. 2011, 2012). Protective *B. longum* strains possessed specific ABC-type carbohydrate transporter that were absent from the non-protective strains. These transporters enabled fructose and mannose utilization in the distal colon, promoting acetate production. In *B. longum* NCC2705, deletion of the fructose-inducible transporter BL0033–BL0036 reduced fructose consumption, acetate production, and protection against lethal EHEC infection (Fig. 3), (Aw and Fukuda 2019). Conversely, introduction of this locus into non-protective *B. adolescentis* JCM 1275^T^ improved survival (Fukuda et al. 2011). These findings demonstrate that the protective phenotype is linked to discrete strain-level genetic traits rather than taxonomic assignment to the genus *Bifidobacterium*.

Furthermore, in mice challenged with EHEC O157:H7, pretreatment with *B. longum* K5 reduced clinical manifestations and colonic inflammation. K5 also attenuated the EHEC-associated increases in serum D-lactic acid and diamine oxidase, suggesting improved intestinal barrier integrity. At the molecular level, K5 restored the expression of tight-junction proteins and MUC2 and reduced the expression of *stx1A* and *stx2A*. Furthermore, K5 reduced the pro-inflammatory cytokines TNF-α and IL-1β while increasing the anti-inflammatory cytokines IL-4 and IL-10 (Tovaglieri et al. 2019, Liu et al. 2024). Collectively, these findings indicate that the protective phenotype of *B. longum* K5 involves coordinated modulation of epithelial barrier function, inflammatory signaling, intestinal microbiota composition, and microbial metabolites, rather than a single mechanism.

Studies using *C. rodentium* provide complementary evidence for strain-specific immunomodulatory effects. Pretreatment with *B. breve* UCC2003 did not prevent *C. rodentium* colonization or A/E lesion formation, but significantly reduced colonic crypt hyperplasia (Collins et al. 2012). Likewise, *B. animalis subsp. lactis* 5764 alleviated *C. rodentium*-induced colitis. Its effects were associated with enhanced IL-17A production by CD4^+^ T cells and NOD2-dependent induction of antimicrobial peptides (Hrdy et al. 2020). Because *C. rodentium* does not produce Shiga toxin, these findings should be interpreted as evidence for modulation of A/E pathogen-associated intestinal inflammation rather than direct protection from toxin-mediated EHEC disease. Other strains have shown protective effects in additional animal models. *Bifidobacterium thermacidophilum* RBL71 reduced fecal EHEC O157:H7 levels, body-weight loss, and intestinal injury in BALB/c mice and increased fecal IgA and serum IgG + IgM responses (Aw and Fukuda 2019). In cell models, cell-free supernatants from *Bifidobacterium lactis* DR10 reduced EHEC O157:H7 counts and invasive ability (Gopal et al. 2001).

Overall, current evidence supports the potential of selected *Bifidobacterium* strains to reduce EHEC-associated intestinal pathology through strain-dependent mechanisms, including acetate-associated barrier protection, reduced Shiga toxin production in selected models, limitation of toxin translocation, direct antagonism in epithelial-cell systems, and modulation of mucosal immune and microbiota responses.

### Lactobacillus

*Lactobacillus* is one of the most extensively studied probiotic bacteria naturally present in the host intestine (Rastogi and Singh 2022). The beneficial effects of *Lactobacillus* are well-proved and include the production of organic acids such as lactic and acetic acids, bacteriocins, and various bioactive metabolites that help maintain gut homeostasis (Lebeer et al. 2008). Specifically, certain strains of *Lactobacillus* have also been reported to enhance gut health by modulating the composition of the microbiota, improving the integrity of the intestinal epithelial barrier, and stimulating host immune defenses (Guo et al. 2017).

Several *Lactobacillus* strains have demonstrated protective effects against EHEC *in vitro* and *in vivo. Lactobacillus rhamnosus* (*L. rhamnosus*) has been shown to protect intestinal epithelial integrity by preventing EHEC-induced disruption of tight junctions, while also reducing EHEC fecal counts in infected mice (Fig. 3), (Hirano et al. 2003, Johnson-Henry et al. 2008, Esfandiari et al. 2025). *Lactobacillus casei* is another strain with antagonistic activity against EHEC. Metabolites produced by *L. casei*, including organic acids, hydrogen peroxide, phenols, and proteins, contribute to its antimicrobial action. These metabolites help eliminate EHEC by competing for resources and adhesion to host cells (Aditya et al. 2020, 2022). Other *Lactobacillus* strains, such as *L. plantarum* and *L. acidophilus*, have also exhibited protective effects against EHEC on the growth and adhesion/invasion and EHEC-induced diarrhea (Campana et al. 2012, Hu et al. 2021, Wang et al. 2023b).

A central role of *Lactobacillus* in combating EHEC is through direct growth inhibition. Multiple strains, including *L. acidophilus, L. johnsonii, L. plantarum*, and *L. rhamnosus*, have demonstrated the capacity to inhibit EHEC growth *in vitro* (Ogawa et al. 2001). In animal models, oral administration of *Lactobacillus* strains before EHEC infection not only reduced intestinal colonization and bacterial survival but also decreased clinical symptoms such as diarrhea, weight loss, dehydration, and intestinal mucosal damage (Kim et al. 2008). This inhibition is attributed to several factors: (1) the production of organic acids (such as lactic acid and acetic acid) lowers gut pH, creating an unfavorable environment for EHEC proliferation; (2) the release of bacteriocins or antimicrobial peptides by *Lactobacilli* can exert targeted inhibition on EHEC; and (3) competition for essential nutrients and further suppresses EHEC colonization (Ogawa et al. 2001, Chen et al. 2007).

Besides inhibiting EHEC growth, *Lactobacillus* strains also inhibit EHEC pathogenicity. Recent studies reveal that *L. acidophilus* La-5 and other *lactobacilli* downregulate LEE genes in EHEC (Zeinhom et al. 2012). Meanwhile, conjugated linoleic acids (CLAs) produced by *L. casei* are potent inhibitors of EHEC toxin expression and secretion (Peng et al. 2020). In particular, *L. acidophilus* has been identified as a key modulator that releases secreted molecules capable of reducing the levels of autoinducer-2 (AI-2), a critical quorum-sensing signal for EHEC to regulate the expression of virulence genes (Medellin-Peña et al. 2007). The ability of *Lactobacillus* species to influence quorum sensing is further supported by molecular studies that demonstrate the downregulation of various transcriptional regulators in EHEC (Medellin-Peña et al. 2007). Consequently, this regulation not only reduces the severity of the infection but may also limit the defense of EHEC to host immune response.

Maintaining epithelial barrier integrity is another fundamental for defense against EHEC. Investigations have demonstrated that specific *Lactobacillus* strains inhibit EHEC-induced disruption of tight junctions, thereby reducing epithelial permeability and preventing the subsequent translocation of both bacteria and toxins (Johnson-Henry et al. 2005). *Lactobacillus rhamnosus* GG, for example, preserves the expression of tight junction proteins such as occludin and zonula occludens-1 (ZO-1) in epithelial cells exposed to EHEC, which significantly restricts the paracellular passage of Shiga toxins (Fig. 3), (Johnson-Henry et al. 2008).

Immunomodulation is another key mechanism leveraged by *Lactobacillus*. The host immune response to EHEC infection is often characterized by an overproduction of proinflammatory cytokines such as IL-8 and an excessive activation of nuclear factor kappa B (NF-κB), which together contribute to tissue damage and disease progression (Stöber et al. 2010, Gao et al. 2013). In HT29 cell co-infection assays, monolayers were simultaneously exposed to EHEC O157:H7 and viable *Lactobacillus* strains. Co-infection with *Lactobacillus rhamnosus* GG, *L. johnsonii* DSMZ 10 533, or *L. reuteri* ATCC 55 730 significantly reduced EHEC-induced IL-8 secretion by HT29 cells, up to about 60% compared with EHEC infection alone (Stöber et al. 2010). This suggests that *Lactobacillus* strains can reduce the inflammatory response triggered by pathogen invasion. By dampening IL-8 levels, *Lactobacillus* helps to limit excessive immune cell infiltration and subsequent tissue damage.

In summary, Recent studies highlight the significant protective effects of various *Lactobacillus* strains against EHEC infection through multiple complementary mechanisms. These findings confirm the important role of *Lactobacillus* in safeguarding the intestinal environment and illustrate their therapeutic potential in the prevention and management of EHEC and other enteric infections.

### Segmented filamentous bacteria (SFB)

SFB are Gram-positive commensal bacteria that colonize the ileal epithelium, characterized by a unique segmented filamentous shape and host specificity. Unlike the majority of commensal bacteria that are spatially separated from the epithelium, SFB directly bind to IECs in the distal small intestine (Ivanov et al. 2009, Ladinsky et al. 2019). SFB closely interact with the ileal villi and Peyer’s patches via their distinctive attachment structures, thereby inducing host cell cytoskeleton remodeling and forming a special adhesion interface (Caselli et al. 2010). Importantly, SFB are predominantly studied in mice and are rarely detected in humans. Therefore, their protective effects should be regarded primarily as evidence from experimental animal models, rather than as a directly translatable human probiotic strategy.

Recent research reveals that SFB can effectively inhibit colonization and infection by *C. rodentium* through the activation of the retinoic acid (RA) signaling pathway in intestinal epithelial cells (Fig. 3). SFB metabolizes dietary vitamin A to produce RA via its own ALDH enzyme, which in turn activates the host’s retinoic acid receptors (RAR). This signaling cascade upregulates key antimicrobial genes, notably *Nos2*, significantly increasing nitric oxide production in the gut. The enhanced NO levels create an inhospitable environment for *C. rodentium*, markedly reducing its survival ability. Notably, this defense operates independently of CD4^+^ T cells, highlighting a unique, microbiota-driven mechanism in which SFB enhances innate epithelial defenses to prevent infection by *C. rodentium*. This finding expands our understanding of how commensal bacteria mediate resistance to gut infections by directly modulating epithelial immunity (Woo et al. 2021). It highlights the potential of dietary vitamin A and microbiota-targeted therapies to enhance intestinal defense against pathogens, which opens the door to potential probiotic or prebiotic interventions aimed at boosting intestinal RA levels to prevent or treat infections. SFB colonize the terminal ileum, however, *C. rodentium* infects the colon. These findings suggest that while SFB do not directly occupy the pathogen’s niche, their ileal activity primes NO-dependent antimicrobial defenses that indirectly restrict *C. rodentium* colonization in the colon.

In addition to RA-dependent epithelial defense, SFB are well known to promote intestinal Th17-cell differentiation, partly through epithelial signals such as serum amyloid A. Th17-associated cytokines, particularly IL-17 and IL-22, can enhance epithelial barrier function and antimicrobial responses, potentially contributing to the later control and clearance of *C. rodentium*. SFB may also enhance mucosal humoral immunity, including secretory IgA responses, which could promote immune exclusion of enteric pathogens by limiting their access to the epithelial surface and reducing adherence. Nevertheless, the relative contributions of Th17 cells, secretory IgA, and RA-driven innate epithelial defenses may vary according to the stage and nature of infection (Woo et al. 2021).

It is important to note that the protective effects of SFB have so far been demonstrated only in animal models, and there is currently no direct evidence that these mechanisms exist in humans. Although direct evidence that SFB inhibit EHEC is lacking, the mechanisms by which SFB protect against C. rodentium provide valuable insights for identifying other bacterial inhibitors of EHEC. In particular, human-associated bacteria or microbiota-derived metabolites that induce epithelial RA signaling, Th17-associated barrier protection, or pathogen-specific secretory IgA may represent promising candidates for strengthening colonization resistance against EHEC.

### Bacteroides thetaiotaomicron

The metabolic interdependence between bacteria in the gut is crucial for understanding the complex interactions between species. *Bacteroides thetaiotaomicron* (*B. thetaiotaomicron*), one of the most abundant anaerobes in the human intestine, plays a central role in the digestion of complex dietary polysaccharides and thereby contributes to host nutrition by releasing monosaccharides (Porter et al. 2018). Through its fermentative metabolism, *B. thetaiotaomicron* produces metabolites such as succinate, fucose, and galacturonic acid, which not only function as nutrient sources for other microbes but also as signaling molecules that influence interspecies interactions. Notably, elevated extracellular concentrations of these metabolites have been shown to modulate the expression of virulence factors in several enteric pathogens, highlighting the potential of *B. thetaiotaomicron* to shape pathogen behavior within the host intestine (Curtis et al. 2014).

First, EHEC utilizes a fucose-sensing two-component system, FusKR, to regulate intestinal colonization (Fig. 3). Fucose, a deoxy-sugar abundant in the host intestine and a component of mucin glycoproteins, is cleaved by microbiota, such as *B. thetaiotaomicron*, from mucin, resulting in high fucose availability in the gut lumen (Gamage et al. 2020, Pearce et al. 2023). The two-component system FusKR, composed of the histidine sensor kinase FusK and the response regulator FusR, enables EHEC to detect these environmental fucose signals and regulate the genes expression accordingly. Within the mucus layer, fucose sensed by the FusKR represses the expression of the LEE, further conserving metabolic resources and promoting efficient colonization. As EHEC migrates toward the intestinal epithelium, where fucose availability declines, the system lifts the repression and activates the adhesion and virulence genes expression. Consistent with this regulatory model, FusK-deficient mutants show markedly reduced colonization efficiency in animal models. Collectively, these observations highlight the pivotal role of FusKR in integrating metabolic cues from the microbiota with the spatial regulation of EHEC virulence during intestinal colonization (Pacheco et al. 2012).

Although *B. thetaiotaomicron* is a well-characterized fucose-releasing commensal, this capacity is not confined to this species. Other *Bacteroides* spp. and certain fucose-utilizing *Bifidobacterium* spp. can metabolize fucosylated host glycans and/or dietary fucosylated oligosaccharides, contributing to fucose cross-feeding in the gut (Pickard and Chervonsky 2015, Kim et al. 2023). This process may enhance colonization resistance by supporting beneficial microbial populations, promoting SCFA production, and limiting pathogen access to ecological niches (Fig. 3). Notably, an engineered *E. coli* Nissle 1917 strain (EcN3) capable of hydrolyzing 2′-fucosyllactose (2-FL) reduced EHEC colonization and intestinal pathology *in vivo* (Ma et al. 2026). The released fucose suppressed EHEC virulence-associated signaling, providing direct evidence that targeted fucose liberation can attenuate EHEC infection. Nevertheless, whether naturally occurring fucose-releasing commensals confer comparable protection requires further investigation.

Recent studies have also elucidated the dual roles of diet-derived galacturonic acid in regulating virulence. It was demonstrated that galacturonic acid, derived from pectin by *B. thetaiotaomicron*, is sensed by EHEC via the ExuR transcriptional regulator (Fig. 3). This molecule serves both as a valuable carbon source, promoting the initial expansion of the pathogen, and as a signal that regulates the expression of LEE genes. The study further extended these findings to *C. rodentium*, demonstrating that galacturonic acid not only promotes pathogen expansion but also regulates the expression of LEE genes, which are essential for establishing intestinal colonization (Jimenez et al. 2020).

Further studies have revealed that *B. thetaiotaomicron* can modulate EHEC virulence by secreting proteases that cleave the T3SS translocon protein EspB, thereby enhancing effector translocation into host cells and promoting A/E lesion formation. Under normal conditions, EHEC itself produces the serine protease EspP, which cleaves EspB in a controlled manner to limit excessive T3SS activity and prevent premature or unregulated effector delivery. In contrast, *B. thetaiotaomicron* secretes distinct proteases that target EspB at alternative cleavage sites and likely at different stages of infection, thereby interfering with EHEC’s temporal control of T3SS activity (Fig. 3). This premature or enhanced cleavage enhances effector translocation and A/E lesion formation, representing the first example of a commensal bacterium directly processing the T3SS components of pathogens. These findings highlight the interaction between microbiota-derived enzymes and pathogen regulatory pathways that can influence infection outcome and disease severity (Cameron et al. 2018).

In conclusion, these findings emphasize the significance of interspecies metabolic interdependence in the host and suggest novel approaches for regulating pathogen virulence by targeting microbiota-derived metabolites and enzymatic activities.

### Enterococcus faecalis

*Enterococcus faecalis* (*E. faecalis*) is a Gram-positive, facultative anaerobic bacterium that belongs to the lactic acid bacteria. *Enterococcus faecalis* inhabits the intestines of healthy individuals as a commensal (Archambaud et al. 2024). However, other reports recognized *E. faecalis* as an important opportunistic pathogen that causes infections, including endocarditis, urinary tract infections, and alcoholic liver steatosis (Duan et al. 2019). Unlike strict anaerobic bacteria, *E. faecalis*, a high tolerance to oxygen levels and can directly colonize the intestinal epithelium (Nishikawa et al. 2009, Nunez et al. 2022). The persistence in the gut is attributed to its ability to survive a wide range of environmental conditions, utilize diverse substrates for energy, and express various factors that promote colonization and survival (Salze et al. 2019).

Recent research shows the complex role of *E. faecalis* in regulating the virulence of EHEC. First, *E. faecalis* significantly enhances the expression of LEE genes. Co-culture experiments have demonstrated that the presence of *E. faecalis* upregulates LEE gene expression and increases the number of EHEC adhering to host cells. Furthermore, purine metabolism and the adenine transporter gene *adeP* are proved to participate in this process. In the absence of *adeP*, the *E. faecalis* induced LEE genes expression is abolished, suggesting that metabolites competition or cross-feeding serves as a regulation mechanism for virulence induction (Martins et al. 2024).

Furthermore, Ethanolamine (EA) is a metabolite derived from the breakdown of phospholipids, such as phosphatidylethanolamine, which is present in both bacterial and eukaryotic cell membranes. The ability to utilize EA as a carbon and nitrogen source is well-recognized among enteric bacteria (Garsin 2012). In *E. faecalis*, enzymes encoded by the *eut* operon mediate EA catabolism, which has been implicated in the survival and competitive fitness of the organism in the intestine (Fig. 3) (Kendall et al. 2012, Luzader et al. 2013). It has been demonstrated that EA not only promotes the growth but also enhances the expression of virulence factors of EHEC (Rowley et al. 2020). The underlying mechanisms by which EA influences bacterial fitness and virulence will be discussed in detail below.

Beyond transcriptional regulation, *E. faecalis* exerts posttranslational modulation of EHEC’s T3SS activity. Specifically, the secreted metalloprotease *GelE* from E. faecalis cleaves EspB, a T3SS effector of EHEC. This proteolytic cleavage enhances effector translocation into host cells, thereby intensifying pedestal formation and facilitating the formation of microcolonies on epithelial surfaces. Mutational analyses confirm that Δ*gelE* of *E. faecalis* fails to augment EspB processing and subsequent T3SS activity (Fig. 3), (Cameron et al. 2019). Importantly, this effect is dependent on direct contact between *E. faecalis* and EHEC, as cell-free conditioned media from *E. faecalis* alone are insufficient to promote T3SS expression (Cameron et al. 2019).

In summary, by influencing both the transcription and activity of EHEC virulence factors through metabolic and proteolytic mechanisms, *E. faecalis* exemplifies the refined roles that the microbiota play in influencing pathogen infection. Understanding these complex relationships is essential for the development of microbiota-based strategies to inhibit intestinal infections.

## Microbiota derived metabolites and EHEC infection

Beyond the specific commensal strains discussed above, a variety of microbiota-derived metabolites also shape the behavior of EHEC in the colon, even if they are not produced by a single, defined bacterial species (Keeney and Finlay 2011, Martins et al. 2022). These metabolites, which include short-chain fatty acids, amino acids, and other metabolites, are produced by diverse gut microbiota. Their presence can influence EHEC virulence by regulating virulence gene expression, signaling pathways, and metabolic adaptation. This highlights the crucial role of the metabolic landscape shaped by the broader microbiota communities, rather than the action of individual strains, in determining EHEC pathogenesis.

### Succinate

Succinate is best known as a component of the TCA cycle, where it is produced from succinyl‐CoA and subsequently oxidized to fumarate by succinate dehydrogenase. Beyond its metabolic role, succinate has garnered significant attention as a signaling molecule in both eukaryotic and prokaryotic organisms (Tretter et al. 2016, Wei et al. 2023). In addition, Succinylation is a chemical modification that alters the charge and structure of proteins, thereby modulating their activity and interactions (Zhang et al. 2011). Enzymes that catalyze Succinylation use succinyl-CoA as the donor of the succinyl group, linking this modification directly to the TCA cycle and metabolic state of the cell (Weinert et al. 2013).

As discussed above, microbial succinate is primarily generated via the fermentation of dietary fibers by certain bacteria, especially those belonging to the *Bacteroidetes* phylum (Connors et al. 2018). In addition, more species are recognized as dominant producers of succinate within the human gut, and the succinate pathway is the main route for propionate formation, a process found in *Bacteroides, Prevotella, Veillonella*, and *Phascolarctobacterium* (Connors et al. 2018). Typically, succinate concentrations in the gut lumen remain low because it is rapidly consumed as an intermediate in propionate production; however, disturbances in microbial community metabolism, such as dietary changes or antibiotic-induced dysbiosis, can lead to the accumulation of succinate (Tsukahara et al. 2003). In bacteria, the succinate levels can reflect shifts in nutrient availability and redox status, and link to cellular processes, including stress responses, biofilm formation, and virulence factor expression (Rosenberg et al. 2021). Several studies have demonstrated that succinate accumulation may serve as a cue for the environmental conditions encountered within the EHEC infection.

**Figure 4:**
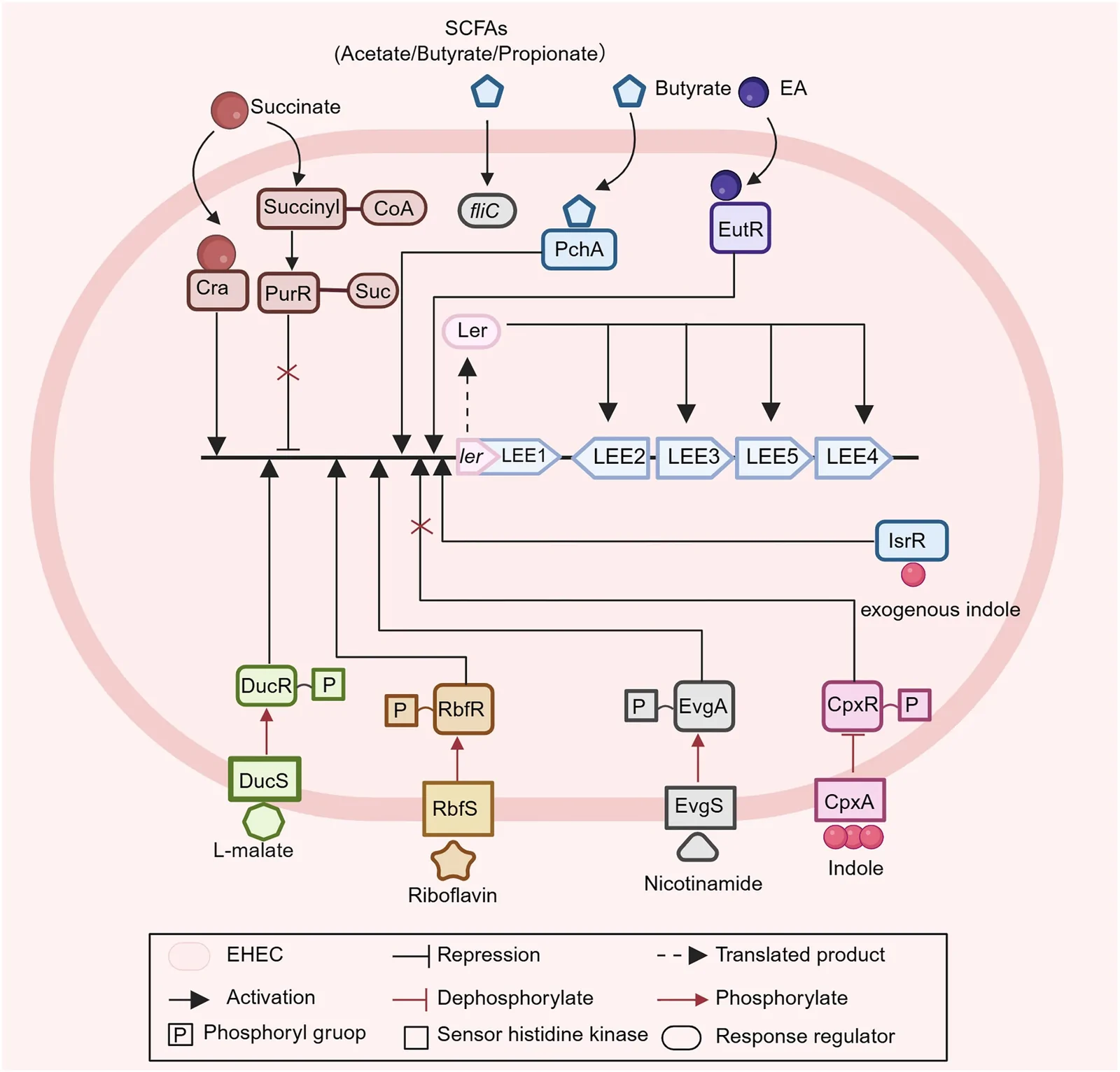
Overview of microbial-derived metabolites influence EHEC virulence by modulating virulence gene expression and signaling pathways. EHEC employs TCS, such as DcuSR, RbfSR, EvgSA, and regulators including IsrR, EutR, and Cra, to sense gut microbiota-derived metabolites to regulate EHEC virulence gene expression.

One of the key regulation pathways through which succinate exerts an influence on EHEC virulence is mediated by the catabolite repressor/activator (Cra) (Fig. 4), (Njoroge et al. 2012). Cra is a global transcriptional regulator that senses intracellular succinate levels and regulates the expression of genes involved in carbon metabolism (Huang et al. 2024). Beyond its classical role in controlling the balance between glycolysis and gluconeogenesis, Cra has emerged as an important regulator of virulence-associated genes in enteric pathogens (Njoroge et al. 2012). In EHEC, studies have shown that the presence of succinate can regulate the expression of key virulence genes by activating the Cra protein. Specifically, succinate may change the DNA-binding affinity of Cra and further alter the expression of target genes. This regulation leads to the enhanced expression of T3SS, and non-LEE encoded (*nleA*, stx2d) virulence genes crucial for EHEC pathogenesis (Curtis et al. 2014).

Recent research has further revealed a mechanism by which host-microbiota interactions regulate the pathogenic potential of *C. rodentium* through succinate signaling. Under normal conditions, group 3 innate lymphoid cells (ILC3s) maintain mucosal homeostasis and restrict the abundance of succinate-producing commensals, such as *Akkermansia muciniphila* (*A. muciniphila*), by limiting intestinal epithelial galactosylation through IL-22-dependent pathways (Fig. 4). When ILC3 or IL-22 function is compromised, galactosylation increases, creating a niche for *A. muciniphila* to expand and produce elevated levels of succinate. This microbiota-derived succinate acts as a potent cue sensed by *C. rodentium*, triggering enhanced expression of key virulence regulators, such as *tir* and *ler*, thus promoting bacterial adherence, colonization, and the severity of infection. This multi-tiered regulation pathways highlights how shifts in the intestinal microenvironment, which is mediated by both host immunity and microbiota composition, can decisively influence the outcome of enteric infections through metabolite-driven modulation of bacterial pathogenicity (Wang et al. 2025b).

Microbiota-derived succinate serves as a key environmental signal that enhances EHEC pathogenicity through a novel posttranslational regulation mechanism. Specifically, succinate produced by commensal bacteria in the large intestine is imported by EHEC and metabolized to succinyl-CoA through the catalytic action of the succinyltransferase CitC, which drives the lysine succinylation of the transcription factor PurR at highly conserved K24 and K55. This Succinylation disrupts the DNA-binding ability of PurR to the promoter of *ler*, relieving PurR-mediated repression and leading to upregulation of LEE gene expression. The result is enhanced bacterial adherence, A/E lesion formation, and colonization in the host. Notably, *in vivo*, studies demonstrate that mice harboring succinate-producing microbiota (e.g. *Segatella copri*) display higher Succinylation of PurR in *C. rodentium*, augmented LEE genes expression, and more severe infection outcomes (Fig. 4). In contrast, depletion or replacement with non-succinate-producing commensals (*Phascolarctobacterium faecium*) abrogates these effects. Thus, microbiota-produced succinate directly influences EHEC virulence by changing the succinylation state, revealing a paradigm for gut microbe–metabolite–pathogen interplay in infection biology (Li et al. 2025).

### SCFAs

Despite the mention of *Bifidobacteria*, SCFAs are predominantly produced in the colon through the anaerobic fermentation of complex carbohydrates and dietary fibers by a wide variety of bacterial species (Miyamoto et al. 2023). Specialized bacterial species, such as *Firmicutes* and *Bacteroidetes*, are primarily responsible for generating these metabolites (Martin-Gallausiaux et al. 2021). Among the SCFAs, butyrate is especially notable for its potent anti-inflammatory properties and its ability to serve as the preferred energy substrate for colonocytes. Acetate, the most SCFAs produced by gut microbiota, serves as a key energy source for host tissues and plays important roles in maintaining epithelial barrier integrity, modulating immune responses, and regulating host metabolic homeostasis (Parada Venegas et al. 2019). Research has increasingly demonstrated that these metabolites play critical roles not only in maintaining host energy balance and gut barrier integrity but also in regulating the virulence of enteric pathogens (Mirzaei et al. 2022).

While high concentrations of SCFAs inhibit EHEC growth, low concentrations of butyrate strongly enhance the expression of virulence genes necessary for bacterial adherence and the formation of A/E lesions. Butyrate, even at very low concentrations (1.25 mM), significantly increases the promoter activity of the LEE1 operon, which controls LEE gene expression through the Ler protein. This effect is dependent on the presence of regulator PchA and requires the leucine-responsive regulatory protein (Lrp). In the distal ileum, where butyrate levels rise, EHEC upregulates its virulence in an Lrp-dependent manner to facilitate colonization. This SCFA-mediated activation highlights the interaction between gut microbiota metabolites and pathogen behavior, suggesting that dietary or probiotic interventions that increase butyrate could inadvertently enhance the pathogenic potential of EHEC (Fig. 4), (Nakanishi et al. 2009).

Beyond the activation of adherence-related genes, SCFAs have been implicated in modulating the motility of EHEC. Research has shown that butyrate, along with other SCFAs such as acetate and propionate, induces flagellar formation in EHEC. However, the underlying regulation mechanisms exhibit a degree of complexity (Tobe et al. 2011). In one experimental model, the DMEM medium, known to enhance virulence gene expression, failed to promote flagellar production on its own (Tobe et al. 2011). In addition, SCFAs regulate flagellar expression to help EHEC adapt to specific gut niches and enhance its pathogenic potential (Lackraj et al. 2016). It has been proposed that EHEC interprets the SCFA gradients encountered along the intestinal tract as spatial cues: small-intestine-like SCFA mixtures markedly up-regulate flagellar genes, FliC production and swimming ability, whereas large-intestine-like mixtures suppress these traits, suggesting a possible mechanism by which EHEC coordinates motility with its localization in the gut.

### Indole and tryptophan-derived metabolites

Indole, a tryptophan-derived small molecule produced predominantly by the gut microbiota, including species from *Bacteroidetes, Bifidobacterium* and commensal *E. coli* via the *tnaA*-encoded tryptophanase (Li and Young 2013, Fang et al. 2022). Notably, indole is unevenly distributed along the gut, reaching its highest concentrations in the lumen, where microbial density is greatest, and declining toward the epithelial surface as it is absorbed by enterocytes (Kumar and Sperandio 2019). By detecting and responding to this spatial gradient, EHEC can fine-tune the timing and location of virulence gene expression during infection.

A key mechanism by which EHEC senses indole involves the CpxA-CpxR two-component system, which modulates envelope stress responses and virulence genes expression (Kumar and Sperandio 2019). CpxA, a membrane-associated histidine kinase, can act either as a kinase or a phosphatase depending on environmental conditions. In the gut lumen, where indole levels are high due to microbiota metabolism, CpxA predominantly acts as a phosphatase, leading to dephosphorylation of the response regulator CpxR (Fig. 4). This prevents CpxR from activating the *ler* promoter, thereby repressing the expression of *ler* and the LEE pathogenicity island. When EHEC adheres to the epithelial cell, indole concentrations decrease, shifting CpxA activity toward its kinase function. This results in phosphorylation of CpxR, activation of *ler*, and subsequent upregulation of LEE genes, which promotes intimate adhesion and A/E lesion formation (Kumar and Sperandio 2019). Interestingly, both indole and the host neurotransmitter serotonin can repress LEE expression through CpxA, but indole uniquely downregulates additional virulence factors, including Stxs, distinguishing its broader regulatory role from that of serotonin (Kumar et al. 2020). Collectively, these findings suggest that EHEC uses the CpxRA TCS to sense indole gradients along the intestine, enabling it to regulate virulence gene expression according to its location within the host.

In addition to the CpxRA-mediated pathway, recent studies have elucidated the role of a novel regulator, IsrR (formerly YgeV), a putative σ54-dependent orphan response regulator, which is likely to control σ54-dependent genes but lacks a cognate sensor kinase, implying a noncanonical signaling mechanism (Fig. 4), (Kumar et al. 2022). In the absence of endogenous indole, IsrR activates LEE gene expression, serving as a critical “on” switch for virulence when indole levels are low (e.g. at the epithelial interface or in microbiota-depleted conditions). Conversely, high levels of exogenous indole derived from the microbiota inhibit IsrR activity and thus repress LEE-dependent pathogenicity (Kumar et al. 2022). This dual regulation enables EHEC to discriminate between environmental sources of indole and finely calibrate its virulence arsenal in response to its precise niche. Studies in *C. rodentium* demonstrate that deletion of isrR leads to reduced intestinal colonization and A/E lesion formation, confirming IsrR’s contribution to *in vivo* virulence (Kumar et al. 2022). Thus, IsrR acts as a key integrator in indole signaling, mediating the pathogen’s adaptation within the host environment.

In addition, microbiota-derived tryptophan metabolites are produced when microbiota metabolize dietary L-tryptophan. These metabolites group includes indole and several indole derivatives, such as indole-3-ethanol (IEt), indole-3-pyruvate (IPyA), and indole-3-aldehyde (I3A), which can function as interkingdom signaling molecules and regulate epithelial and immune responses (Roager and Licht 2018). In addition to directly modulating EHEC virulence discussed above, these metabolites can promote colonization resistance through host-directed signaling pathways.

Recent research showed that IEt, IPyA, and I3A activate dopamine receptor D2 (DRD2) in intestinal epithelial cells. These metabolites reduced the attachment of EHEC to intestinal epithelial cells and protected mice against infection with the A/E pathogen *C. rodentium*, a widely used surrogate model for EHEC infection. Mechanistically, IEt, IPyA, and I3A did not directly alter pathogen growth or virulence-factor expression. Instead, activation of epithelial DRD2 triggered a Gβγ–PLC–PKCθ signaling cascade, promoted WIP phosphorylation, and reduced the abundance of N-WASP, a host actin-regulatory protein hijacked by attaching-and-effacing pathogens for actin-pedestal formation. Consequently, DRD2 activation limited pathogen attachment and intestinal colonization. Genetic deletion of *Drd2* in intestinal epithelial cells abolished the protective effects of the tryptophan diet and microbial metabolites, whereas pharmacological DRD2 activation reproduced these effects (Scott et al. 2024). Dopamine receptor D2 confers colonization resistance via microbial metabolites.

In conclusion, these works expand the current view of indole signaling at the host-microbiota-pathogen interface. Whereas luminal indole can directly repress EHEC virulence through bacterial sensing systems such as CpxRA and IsrR, specific microbiota-derived tryptophan metabolites can also act on epithelial DRD2 to restrict the host cytoskeletal processes required for efficient A/E lesion formation.

### Riboflavin

Riboflavin is an essential micronutrient that serves as a precursor to the coenzymes flavin mononucleotide (FMN) and flavin adenine dinucleotide (FAD) (Powers 2003, Wang et al. 2025a). These coenzymes are indispensable in various redox reactions that sustain energy metabolism and cellular homeostasis. Gut microbiota routinely produce riboflavin, and its levels in the intestinal lumen can significantly influence the behavior of resident or invading bacteria. In the human gut, riboflavin emerges as a crucial signaling molecule, owing to its dual role in both nutrition and signaling (El Morr et al. 2024).

In particular, the riboflavin-sensing mechanism mediated by the TCS Riboflavin Sensor and Regulator (RbfSR) in EHEC highlights a strategic adaptation that enables the pathogen to regulate its virulence in response to microbiota-derived signals. The autophosphorylation of RbfS induces the process underlying riboflavin sensing in EHEC. Once phosphorylated by RbfS, the activated RbfR significantly enhances its binding to the promoter region of the LEE operon (Fig. 4). The binding of phosphorylated RbfR to the *ler* promoter stimulates the transcription of LEE genes. This upregulation of virulence determinants facilitates bacterial adherence, *in vivo* colonization, and the severity of the disease (Liu et al. 2022).

### Nicotinamide

Nicotinamide, also known as niacinamide or nicotinic acid amide, is the amide form of vitamin B3 (niacin) and plays an essential role in cellular metabolism. As a precursor for nicotinamide adenine dinucleotide (NAD⁺), nicotinamide is important for energy production, oxidative phosphorylation, and several metabolic reactions that sustain cellular homeostasis (Bogan and Brenner 2008). It is synthesized endogenously and can also be derived exogenously from dietary sources, as well as from the microbiota (Uebanso et al. 2020). Notably, the high abundance of nicotinamide in the intestinal lumen, combined with its efficient absorption through the epithelial layer (Niño-Narvión et al. 2023). In the host, nicotinamide contributes to NAD⁺ biosynthesis, regulating energy metabolism, mitochondrial function, and sirtuin–dependent anti–inflammatory pathways that support epithelial barrier integrity and immune homeostasis (Niño-Narvión et al. 2023). At the microbial level, nicotinamide availability influences bacterial NAD⁺ metabolism, growth, and redox balance, ultimately shaping community composition and the production of metabolites that impact intestinal inflammation and host physiology (Niño-Narvión et al. 2023). So, the interplay between nicotinamide and the resident microbiota has profound health implications.

The regulation of EHEC O157:H7 virulence by nicotinamide is orchestrated through the activation of the TCS EvgSA, a highly conserved regulatory pathway among various bacterial lineages. In detail, EvgS functions as the membrane-bound sensor kinase, while EvgA acts as the cytoplasmic response regulator (Yang et al. 2023). Upon exposure to elevated levels of nicotinamide in the large intestine, EvgS undergoes autophosphorylation. After autophosphorylation, the phosphoryl group is transferred from EvgS to EvgA, thereby activating the response regulator. Activated EvgA directly interacts with the promoter region of the *ler*, which subsequently activates the transcription of downstream LEE operons. The resultant increase in transcription of genes encoding T3SS components and various effectors culminates in enhanced bacterial adherence to host cells and the formation of pathogenic A/E lesions (Fig. 4), (Yang et al. 2023).

### Ethanolamine

Ethanolamine (EA) is a ubiquitous compound derived from the breakdown of phospholipids, a principal constituent of both bacterial and mammalian cell membranes. It is released into the intestinal lumen following cell turnover and exfoliation, serving not only as a valuable nutrient source but also as a signaling molecule that can influence gene expression in microbes, notably activating the *eut* operon (*eutB, eutC, eutE, eutR*) involved in ethanolamine metabolism and modulating virulence genes such as the LEE genes and *stxs* in EHEC (Tsoy et al. 2009, Garsin 2010).

From a metabolic perspective, EA serves as a key source of carbon and nitrogen. Bacteria possess the EA utilization (*eut*) operon that can catabolize EA to generate vital precursors for biosynthesis and energy production (Marshall et al. 2023). Beyond its metabolic contributions, EA has been recognized as a signaling molecule that regulates gene expression. It is noteworthy that EA metabolism in many bacteria, including EHEC, is closely linked to the availability of vitamin B12 (cobalamin). Vitamin B12 acts as a cofactor for several enzymes within the *eut* pathway, facilitating the conversion of EA. The interdependency of EA and vitamin B12 highlights a sophisticated mechanism whereby nutrient availability has a direct impact on virulence (Garsin 2010). As discussed above, studies have shown that the interaction between EA and vitamin B12 plays a crucial role in the activation of EutR and subsequent virulence regulation (Rowley et al. 2020). When EA is abundant, the EutR binds to EA and enhances its binding ability to the promoter regions of the *eut* operon. Vitamin B12, serving as a critical coenzyme, further enhances the activation of EutR. Activation of EutR has been correlated with the differential expression of LEE genes and Shiga toxins in EHEC. This regulation pathway supports the connection between the metabolic state of the pathogen and its ability to form A/E lesions (Fig. 4), (Bertin et al. 2011, Rowley et al. 2020).

Taken together, the mechanistic insights into EA regulation in EHEC emphasize the complexity of bacterial adaptation within the host environment. By utilizing EA as both a nutrient source and a signal, EHEC employs a novel strategy to modulate its metabolic and pathogenic capabilities.

### L-malate

L-malate is a naturally occurring C4-dicarboxylic acid that plays an important role in cellular metabolism as an intermediate in the TCA cycle (Nguyen et al. 2020). Furthermore, the importance of L-malate extends beyond energy metabolism; it also serves as a precursor in several biosynthetic pathways, including amino acid (e.g. aspartate) and nucleotide synthesis, gluconeogenesis, and redox balance (Unden et al. 2016).

Recent research has shown that L-malate is central to the pathogenesis of EHEC, serving not only as an essential nutrient for anaerobic respiration but also as a critical signal for regulating virulence gene expression. First, L–malate emerges as a substrate due to its abundance in the colon and its efficient uptake by EHEC. Notably, EHEC utilizes a set of transporter proteins (DcuABC) to import L-malate from the intestinal lumen, both as a metabolite supporting fumarate respiration and as a signaling molecule that activates the LEE genes expression, thereby enhancing intimate adherence to the host epithelium (Fig. 4). *In vivo*, mutants defective in L–malate transport (e.g. Δ*dcuB*) or metabolism exhibit significant colonization defects compared to the wild–type strain. In particular, the Δ*dcuABC* mutant exhibits an approximately 90-fold reduction in colonization efficiency, underscoring the importance of L-malate uptake and utilization in EHEC’s survival within the host intestine (Liu et al. 2023).

In parallel with its role in metabolism, L–malate also functions as a signaling molecule that directly regulates the expression of virulence genes in EHEC. Upon entering the large intestine, the sensor kinase DcuS senses elevated L–malate levels and becomes auto-phosphorylated. The phosphate is then transferred to the response regulator DcuR(DcuR–P). Activated DcuR–P binds directly to the promoter region of the *ler*. This transcriptional activation by DcuR–P ensures that EHEC upregulates LEE genes and A/E lesions (Liu et al. 2023).

In summary, the role of L–malate in both energy metabolism and virulence regulation represents an example of how EHEC integrates environmental cues to enhance the pathogenic processes. This emerging understanding offers exciting opportunities for the development of targeted therapies designed to disarm EHEC’s virulence without compromising host welfare.

### Translational relevance of microbiota-derived metabolites

The translational relevance of microbiota-derived metabolites stems from their dynamic and spatially heterogeneity. Such dynamics is shaped by differences in microbial community composition and functional capacity, host genetics, age, intestinal transit time, epithelial physiology, and dietary patterns (Zierer et al. 2018, Johnson et al. 2019). Importantly, metabolite concentrations are not uniform throughout the intestine and may differ between the lumen and mucus layer, as well as between proximal and distal intestinal regions (Tropini et al. 2017). This spatial heterogeneity is particularly relevant to EHEC infection, as the pathogen encounters distinct nutrient and signaling environments while traversing the intestine and approaching the epithelial surface (Pacheco et al. 2012, Donaldson et al. 2016).

Diet represents one of the most accessible and modifiable determinants of this metabolic environment. Diets rich in complex carbohydrates and dietary fibers generally promote saccharolytic fermentation and the production of SCFAs, particularly acetate, propionate, and butyrate (Makki et al. 2018). These metabolites can contribute to the maintenance of epithelial barrier integrity, mucus production, immune regulation, and suppression of pathogen expansion under certain conditions. In contrast, dietary protein and amino acid availability may influence the production of metabolites derived from amino acid catabolism, including indole and other tryptophan-derived compounds (Roager and Licht 2018, Agus et al. 2021). The intake of vitamins and vitamin precursors may additionally affect the availability of metabolites such as riboflavin and nicotinamide, either directly or through their effects on microbial metabolic activity (LeBlanc et al. 2013). Thus, dietary composition may influence EHEC colonization resistance both indirectly, by reshaping microbial community composition, and directly, by altering the concentration and spatial distribution of pathogen-relevant metabolites. However, a beneficial metabolic profile cannot be defined simply by increasing or decreasing a single compound. Instead, the overall balance of metabolites, their spatial distribution, and their interactions with the resident microbial community should be considered.

Disease can further reshape the intestinal metabolite landscape. Intestinal inflammation, antibiotic exposure, diarrheal disease, and microbiota disruption can alter microbial community composition, oxygen availability, epithelial metabolism, and nutrient release (Winter et al. 2013). These changes may affect both concentrations and local accessibility of microbiota-derived metabolites. For example, inflammation-associated alterations in intestinal oxygen availability may favor the expansion of facultative anaerobes and subsequently alter the metabolic output of the gut microbiota (Rivera-Chavez et al. 2016). Likewise, epithelial damage or increased mucus turnover may release host-derived nutrients that can be exploited by enteric pathogens (Pacheco et al. 2012). Consequently, disease-associated metabolic changes may weaken colonization resistance not only by reducing protective metabolites but also by generating nutritional conditions that support EHEC growth, virulence gene expression, or persistence. Nevertheless, direct longitudinal evidence linking specific metabolite levels with the outcomes of human EHEC infection remains limited.

Collectively, these findings support the view that microbiota-derived metabolites are modifiable determinants of colonization resistance rather than immutable signatures of a given microbiome. Potential strategies to manipulate this metabolic landscape include dietary fiber supplementation, targeted nutrient or vitamin supplementation, prebiotics, probiotics, or defined microbial consortia with specific metabolic functions, and microbiota-based interventions such as fecal microbiota transplantation. However, interventions aimed at modifying a single metabolite may have unintended effects on pathogen behavior, commensal microbial ecology, or host physiology. Future studies should therefore integrate metabolomics, metagenomics, and host clinical data to identify metabolite profiles consistently associated with resistance or susceptibility to EHEC colonization. Such integrated approaches may facilitate the development of personalized dietary and microbiome-targeted strategies that enhance colonization resistance while minimizing the risk of promoting pathogen virulence.

## Potential EHEC treatment strategy through microbiota

**Figure 5:**
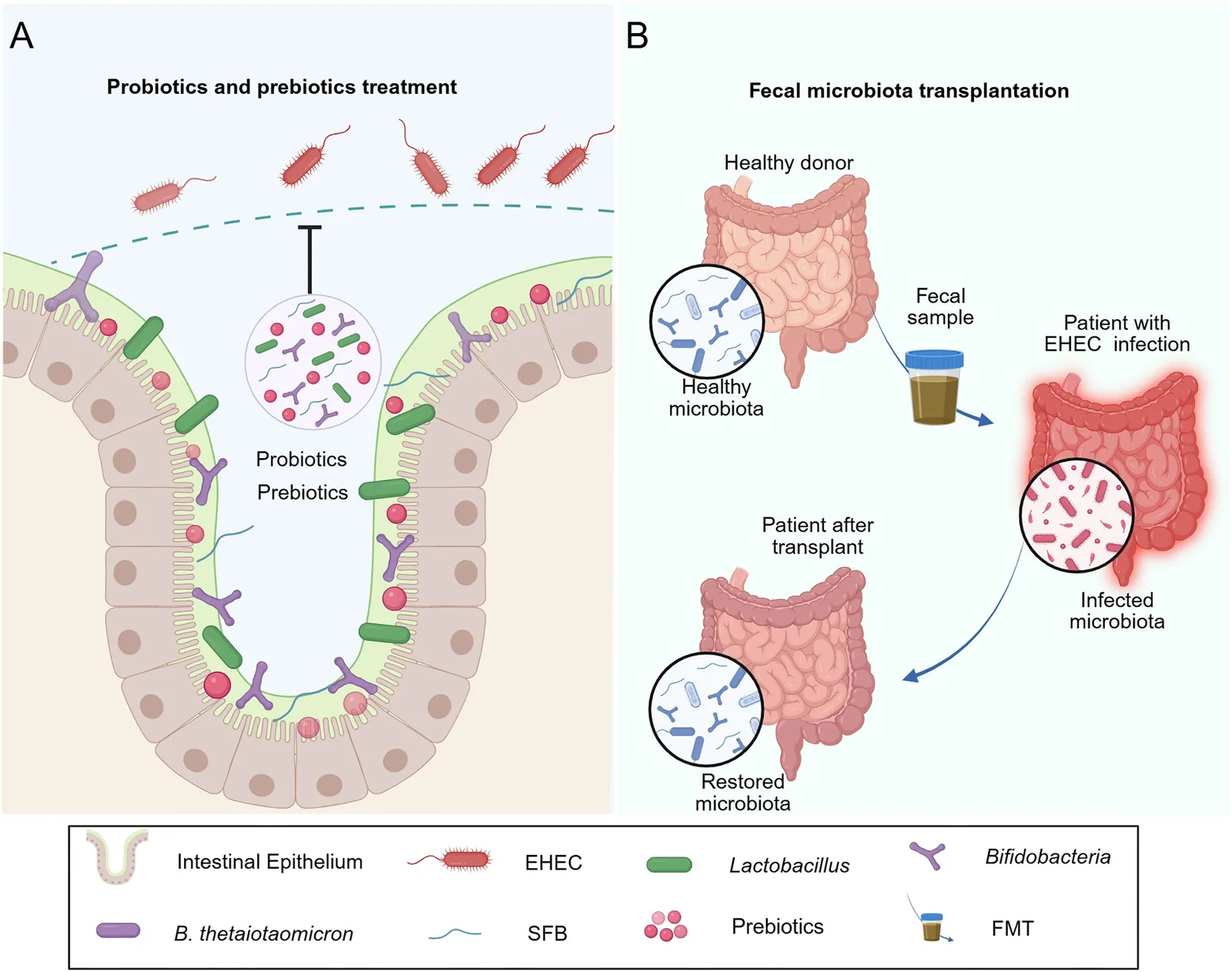
Microbiota-targeted strategies in controlling EHEC infection. (A) Probiotics and prebiotics inhibit EHEC infection. Block EHEC colonization via competitive adhesion to intestinal epithelium. Repress EHEC virulence genes through microbiota modulation. Attenuate virulence by modifying host-microbiota interaction. (B) Fecal microbiota transplantation suppresses EHEC infection. Reconstitutes diverse microbiome to limit EHEC expansion.

The recent focus on manipulating gut microbiota offers a promising avenue for the prevention and treatment of intestinal infections (Kamada et al. 2013). Conventional antimicrobial therapies can effectively treat infections caused by pathogens, but their broad application tends to interfere with the diversity and roles of the resident microbiota (Bäumler and Sperandio 2016). Such dysbiosis might cause problems including colitis associated with antibiotics and the rise of antimicrobial resistance (Cabral et al. 2019, Khan et al. 2021). Alternatively, microbiota-centered approaches, like probiotics, prebiotics, and fecal microbiota transplantation (FMT), present new alternatives by aiming to restore microbial balance, enhance the gut barrier, and modulate the host immune responses (Fig. 5), (Chen et al. 2024).

First, several studies have demonstrated that probiotics offer significant potential in preventing and reducing colonization by EHEC (Sherman et al. 2005). Certain probiotic strains can combat EHEC through different mechanisms. Initially, by attaching to intestinal epithelial surfaces, probiotics competitively block pathogen attachment and prevent EHEC colonization (Johnson-Henry et al. 2007). Moreover, probiotics have the potential to influence the host environment and microbiota, inhibiting the expression of virulence genes, especially the formation of A/E lesions (Medellin-Peña and Griffiths 2009). The dual function of targeted probiotic therapy, blocking initial colonization and attenuating key virulence traits, emphasizes its therapeutic potential for mitigating the clinical impact of EHEC infection. Furthermore, the application of prebiotics serves as an extension of microbiota modulation strategies. *In vitro* and *in vivo*, prebiotics selectively stimulate the growth of commensals that inhibit EHEC growth and pathogenicity (Tabashsum et al. 2019). Both administered with prebiotics and probiotics, prebiotics enhance the survival and efficacy of probiotics, enhance the restoration of microbial diversity, and the function of intestinal barrier, resulting in a collaborative therapeutic outcome (Tabashsum et al. 2019). Significantly, such strategies can decrease the colonization niches of EHEC and reduce the immune activation of pathogens (Khan et al. 2021).

Fecal microbiota transplantation (FMT) has become an established therapy in the management of pathogen infections, such as *Clostridioides difficile, Listeria monocytogenes*, and EIEC (Oksi et al. 2020, Khan et al. 2021, Guo et al. 2023, Wang et al. 2023a). However, its efficacy against EHEC infection remains poorly understood. Although direct evidence from EHEC studies is limited, findings from experimental models of EIEC infection suggest that FMT may exert protective effects by restoring intestinal microbial balance and inhibiting pathogen colonization (Fig. 5), (Wang et al. 2023b). Based on these mechanisms, FMT represents a promising therapeutic strategy that could potentially be applied to EHEC infection. Nevertheless, further clinical investigations are required to validate its safety and efficacy of EHEC infection.

Collectively, microbiota-based interventions, including probiotics, prebiotics, and FMT, offer a multifaceted and targeted approach for controlling EHEC infection and restoring gut homeostasis. Further research is warranted to clarify the optimal strategies and elucidate the underlying mechanisms that support their clinical efficacy.

## Current challenges and future research prospects

Microbiota–targeted interventions have shown encouraging results in animal models, yet several hurdles must be overcome before transition to clinical use. Unlike laboratory animals, humans exhibit strong inter–individual variation in gut microbiota composition and metabolism, making therapeutic outcomes highly variable and difficult to predict (Chen et al. 2024). Current evidence is also mostly short term, and the long–term safety and durability of these approaches remain poorly characterized. Meanwhile, a deeper mechanistic understanding is still lacking at the molecular and cellular levels. Specifically, how pathogens exploit microbiota–derived metabolites, how inflammation remodels the intestinal metabolic landscape, and how nutrient competition influences virulence and colonization.

To overcome these challenges, comprehensive microbiome profiling integrated with multi–omics (genomics, transcriptomics, proteomics, and metabolomics) is needed to design individualized interventions (Yan et al. 2020). Combining metagenomic and metabolomic data enables the reconstruction of microbe–metabolite–host networks that link microbial taxa to functional metabolites (Robinson et al. 2019, Sun et al. 2024b). Through this systems–level analysis, the microbiota and metabolic nodes may be identified as potential targets that regulate EHEC virulence and host immunity. Moreover, the microbiota dysbiosis across infection stages are poorly defined. Longitudinal multi-omics sampling coupled with functional validation will be essential to capture the spatiotemporal dynamics of microbiota and metabolic alterations and to identify stage-specific driver microbes and metabolites.

Leveraging artificial intelligence (AI)–driven modeling and causal inference of microbe–metabolite–host interactions can accelerate the design of microbiota–targeted therapies (Zhang et al. 2024, Rozera et al. 2025). By integrating multi–omics and clinical data, machine learning–based network reconstruction can identify critical metabolites and regulatory pathway that predict host responses and pathogenic process. AI–derived insights can support the rational design of next–generation combination therapies, integrating probiotics or synbiotics with targeted metabolic modulation, to restore colonization resistance and attenuate LEE–dependent virulence.

In conclusion, precise patient stratification, deep multi–omics integration, AI–guided modeling, and longitudinal mechanistic mapping are key to bridging the gap from effective in animals to clinical use. By targeting actionable commensals and metabolites, microbiota–based strategies can be developed into intelligent and personalized interventions that enhance colonization resistance and suppress pathogen virulence, thereby facilitating their clinical translation for EHEC control.

## Data Availability

All data generated or analyzed during this work are included in this published review.
